# Education and social care predictors of offending trajectories: A UK administrative data linkage study.

**DOI:** 10.23889/ijpds.v7i3.1928

**Published:** 2022-08-25

**Authors:** Hannah Dickson, George Vamvakas, Nigel Blackwood

**Affiliations:** 1 King's College London

## Objectives

Total annual costs of crime in England and Wales is estimated at £50bn.The age-crime curve indicates that criminal behavioural peaks in adolescence and decreases in adulthood. Life-course persistent offenders begin to behave antisocially early in childhood and continue this behaviour into adulthood. By contrast, adolescent-limited offenders exhibit most of their antisocial behaviour during adolescence, with a minority continuing to offend into adulthood. However, evidence suggests that this curve conceals distinct developmental trajectories. Prospective cohort study data has highlighted distinct risk factors for these offending trajectories, but this research is limited because of small sample sizes for disadvantaged groups, selection bias and infrequency of data collection.

## Approach

The current study began in February 2022 and is one of the first to use UK linked national crime and education records. The aim is to: (1) establish the offending trajectories of individuals between the ages of 10 and 32 years following their first recorded conviction or caution using national crime records; and (2) develop prediction models of these offending trajectories using administrative education and social care data.

## Results

In my talk, I will share findings on the offending trajectories identified and present some early results on the key education and social care drivers of the offending trajectories.

## Conclusion

Findings from the project have the potential to identify previously unknown, or confirm lesser known, offending trajectories using real world data based on the UK population. It may also lead to the detection of previously unknown risk or protective factors for offending, which has implications for early intervention and could help inform criminal justice system responses to early antisocial behaviour.

